# Breathing therapy for patients with medically unexplained physical symptoms and dysfunctional breathing: A pilot and feasibility trial

**DOI:** 10.1371/journal.pone.0325951

**Published:** 2025-07-11

**Authors:** Hege Svenningsen, Trine Stub, Rosalba Courtney, Tor-Ivar Karlsen

**Affiliations:** 1 Lege Hege Svenningsen AS, Norway; 2 UiT The Arctic University of Norway, Department of Community Medicine, Faculty of Health Science, Norway; 3 Southern Cross University, Lismore, New South Wales, Australia; 4 University of Agder, Department of health and nursing, Norway; Maulana Azad Medical College, INDIA

## Abstract

Medically unexplained physical symptoms (MUPS) are symptoms without an identifiable organic cause that lead to functional impairment. MUPS is highly prevalent in general practice consultations. This pilot trial aimed to investigate the effectiveness and feasibility of a 5-week breathing retraining program focusing on basic anatomy and physiology, breathing awareness, nasal breathing and resonance (coherent) breathing for patients meeting the criteria for MUPS.

The trial used a quantitative design with pre- and post-intervention measurements. Fifteen participants with MUPS and dysfunctional breathing (assessed by the Nijmegen Questionnaire) were recruited from two general practitioner offices. The intervention consisted of 5 weekly sessions including education on breathing physiology and weekly breathing exercises focused on nasal breathing and resonance breathing techniques. One week post intervention, improvements were observed in dysfunctional breathing scores, lower symptom severity, higher general well-being, and reduced musculoskeletal pain complaints. At 3 months post-intervention, sustained improvements were seen in dysfunctional breathing, general well-being, musculoskeletal pain, and additionally lower pseudoneurological, gastrointestinal, and allergy complaints, as well as lower overall symptom burden and improved end-tidal CO_2_ levels. The trial concluded that the 5-week breathing program showed promising results for improving multiple patient-reported outcomes in MUPS. Recruitment, adherence, and acceptability of the program were satisfactory. A randomized controlled trial is recommended to further evaluate the efficacy of this breathing intervention for MUPS patients. Trial registration: ClinicalTrials.gov NCT06575920

## Introduction

Medically unexplained physical symptoms (MUPS) encompass somatoform conditions characterised by symptoms without corresponding objective findings. MUPS is thus an umbrella term that refers to conditions characterised by multiple, co-occurring chronic physical symptoms [1]. We distinguish between daily and self-limiting complaints and persistent MUPS, which involve long-lasting symptoms and loss of function [2]. Although the term is debated, it is considered appropriate for clinical and research purposes as there are substantial similarities and comorbidity across different MUPS disorders, indicating possible common underlying symptom mechanisms. Other descriptions mentioned in health research literature include bodily distress syndrome/disorder, somatisation and functional somatic symptoms disorder [1,3,4].

A literature review of research on MUPS found international prevalence rates ranging from 2.9% to 76% in high users of primary healthcare, with most studies finding prevalence rates ranging from 20% to 50% [5–7]. It is estimated that up to 40% of all consultations in primary care involve patients presenting with MUPS [5]. Additionally, due to the high number of referrals and further examinations, these patients are also commonly encountered in specialist healthcare [5].

MUPS is highly prevalent across all healthcare settings and accounts for approximately 45% of all general practice consultations [6] and 20% of new consultations in primary care [8]. In a Norwegian survey from 2017, general practitioners (GPs) registered 526 MUPS patients among their total of 17,688 consultations, giving a consultation prevalence of persistent MUPS of 3%. Musculoskeletal pain and asthenia/fatigue were predominant symptoms, affecting 68.1% and 57.0% respectively. Headache or dizziness was reported by 31.9% of the patients, and gastrointestinal symptoms by 20.5% [2].

The costs associated with MUPS are substantial, as patients undergo extensive investigations in both primary and specialist health services. Most of the sick leave and disability in Norway and other Western countries is claimed to be caused by MUPS [9]. A literature review from 2012 showed that MUPS causes relevant annual costs in healthcare that are comparable to mental health problems like depression or anxiety. Multiple MUPS are associated with negative treatment outcomes in other disorders [10].

A Cochrane review on pharmacological interventions for MUPS found very low-quality evidence for antidepressants and natural products being effective in treating somatoform symptoms in adults when compared with placebo [11]. Although cognitive behavioural therapy (CBT) has demonstrated effectiveness in specialised settings, its efficacy in primary care has been relatively limited [12]. The implementation of the structured communication tool in primary care in Norway significantly improved patient outcomes and reduced sick leave among patients with MUPS [5].

In a Norwegian study, GPs reported that supportive counselling was the most common management strategy [9]. In a paper on the lack of evidence for effective treatments of MUPS, the authors state that ‘for real progress to advance, new questions may be more crucial than old answers’ [13].

With a respiration rate of 15 breaths per minute, we breathe approximately 22,000 times a day. It might be reasonable to think that the way we breathe affects our health in the same way as how we eat, sleep and move. Considering the bidirectional relationships of breathing with multiple body systems and its central role in homeostasis [14], questions arise regarding the relationships between the symptoms of MUPS and sub-optimal or dysregulated breathing.

The term Dysfunctional Breathing (DB) is commonly used to describe a range of disturbances in breathing regulation and control that impact symptoms and quality of life [15]. It has also been defined as a condition where breathing is maladaptive and does not efficiently perform its various functions [16]. The development of DB can be primary and not explainable by organic disease or secondary to specific respiratory conditions [17]. It has also been linked to increased allostatic load where abnormal pattern of breathing, which may begin as a “coping mechanism” to deal with periods of stress become habitual [15]. While DB is not precisely defined, its characteristics, impacts, assessment, and treatment have been frequently described in the literature [18,19]. The prevalence of DB among adults in primary care in the United Kingdom is approximately 9.5%, being most prevalent among women and asthmatics [19].

According to the scientific literature, DB patterns might be harmful, self-perpetuating patterns [14,20] and may partially explain some MUPS [18,21,22]. There are indications that its correction after breathing therapy combined with stress reduction and lifestyle management is associated with reduced symptoms and improved quality of life [23–25]. Breathing therapy may also be beneficial for somatic syndromes, such as irritable bowel syndrome [26], fibromyalgia and ME/CFS [27] and post-traumatic stress disorder [20]. Brain imaging studies reveal breath-activated pathways to all major networks involved in emotion regulation, cognitive function, attention, perception, subjective awareness and decision-making [20].

Breathing therapy involves voluntary changes in the rate, pattern and quality of respiration [20]. The main rationales for breathing therapies include: 1) correcting some aspect of DB; 2) supporting one or several functions of breathing and thus stimulating healing; or 3) providing a means for stabilising mental and emotional states [14].

Breathing therapy has shown positive results on various individual unexplained symptoms including respiratory discomfort or dyspnoea with ‘air hunger’ or a sense of unsatisfied respiration [22], dizziness, peripheral neurovascular symptoms such as tingling and numbness [27], back and neck pain [21,28], fatigue [29,30], headache [31,32] and inner turmoil [31]. If shown to be effective in patients with MUPS, breathing therapy could be used in appropriate patients alongside other evidence-based treatments [5]. While breathing therapy is an easy and low-cost modality to implement, more research, especially randomized controlled trials (RCT) are needed to establish its effectiveness and refine its application in a general practice setting in a group of patients with MUPS and DB.

Our basic hypothesis is that correcting DB patterns would reduce MUPS severity. In order to establish the grounds for a RCT this pilot trial aims to investigate the effectiveness and feasibility of a 5-week breathing retraining programme on patients who meet the criteria for MUPS.

## Methods

### Design

The pilot and feasibility trial employed a quantitative pre-test and post-test design to evaluate the feasibility of a breathing therapy protocol (recruitment rates, adherence, and acceptability), assess patients’ experiences and measure the extent of symptoms pre- and post-breathing therapy. The design facilitated statistical analysis of the data using established statistical methods [33].

### Ethical considerations

The trial received approval from the Norwegian Regional Committee for Medical and Health Research Ethics (ref 590376) and The Research Ethics Committee at the Faculty of Health and Sports at the University of Agder (ref RITM0234474). Data protection was assessed and approved by the Norwegian Agency for Shared Services in Education and Research (Sikt) (ref 840311).

Relevant and anonymized data can be accessed from Sikt – Norwegian Agency for Shared Services in Education and Research [34].

Participants were briefed on the trial and each of them provided informed consent. Due to the limited number of study participants and the nature of this pilot and feasibility trial, the trial was registered in the ClinicalTrials database (ref NCT06575920) after recruitment of study participants. The authors confirm that all ongoing and related trials for this intervention are registered

HS received a grant of NOK 200.000 (appr. Euro 20.000) from the Regional Research Foundation, County of Agder, Norway (ref. 961571). The funder did not influence the designe, data collection, analyses, decision to publish or preparation of the manuscript.

### Intervention

The 5-week breathing therapy intervention, developed by the first author, was based on Rosalba Courtney’s “Integrative Breathing Teacher Training” [14,17], relevant research, and the first author’s clinical experience. The sessions were conducted by the first author, an experienced general practitioner trained in Integrative Breathing Therapy and yoga teaching.

Each 90-minute session included basic education on respiratory anatomy and physiology, guided breathing exercises, and time for reflection on experiences, questions, and planning of home practice. Participants were encouraged to complete 20 minutes of daily breathing exercises during the intervention period. These 20 minutes could be divided into shorter sessions. Informal breathing practice was encouraged but optional.

The intervention focused on nasal breathing and coherent (resonance) breathing, two approaches with documented beneficial results [35–39]. Nasal breathing was emphasised as the nose is a highly specialised respiratory organ that, among other functions, moistens, tempers and cleanses the air, and has several health-supporting effects. Coherent breathing was introduced briefly already the first session, and progression was made with applying focus on nasal breathing, muscular function, knowledge of the autonome nervous system and the role of CO_2_. Each session adding knowledge and exercises connected to theory, mainly with bases in resonant breathing and nasal breathing. Coherent or resonance breathing is defined as gentle breathing with equal duration of inspiration and expiration at approximately 5–6 breaths per minute, a rate that optimally balances sympathovagal stress response for most adults [20]. Coherent breathing occurs when oscillations from the heart and breathing synchronise or become resonant. This typically results in the highest levels of heart rate variability (HRV), which is used as an indicator of autonomic nervous system functioning [23,39,40].

Sessions 2–5 were held at a yoga center. Session 1-, 6- and three-months post intervention follow up were held at a private home.

### Inclusion and exclusion criteria

Patients were included if they were 18 years of age or older and met the criteria for MUPS with DB. MUPS is defined as physical symptoms without an identifiable organic cause, lasting for at least three months and leading to loss of function. Loss of function is explained as illness, absence or disability, or withdrawal from social activities. DB was defined as a Nijmegen Questionnaire (NQ) score ≥20 (the NQ is validated to measure the degree of DB) [41]. Additional inclusion criteria were the ability to fill in a consent form and sufficient knowledge of Norwegian to understand the questionnaires accurately. Participants with asthma, chronic obstructive pulmonary disease, or respiratory allergy that is not optimally treated medically were excluded from participation.

### Recruitment

**Fig 1:**
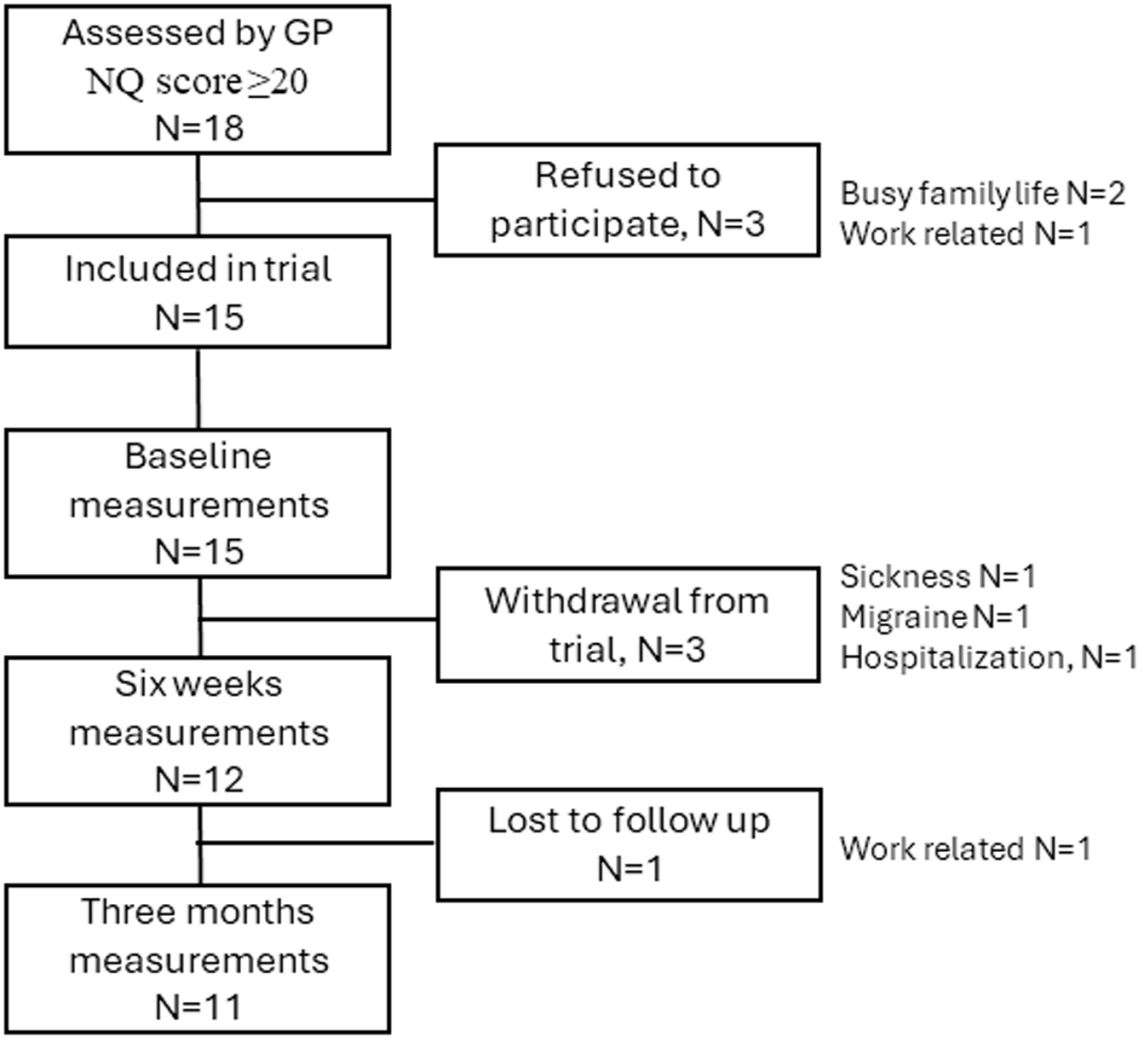
Trial flowchart.

Between 01/09/2023 and 30/09/2023, patients with one or more MUPS who visited two local GP offices received an information leaflet on the trial. Patients who met the inclusion criteria were asked by the GP if they wanted to participate in the trial and, if accepted, their contact information was sent to the researcher (N = 18). At the first session, the researcher informed participants of the content and duration of the intervention programme and collected signed informed consent from a total of 15 participants. A flowchart for the trial is presented as Fig 1. Participants were divided into two groups, one meeting in the daytime and the other in the evenings, according to their preference.

### Data collection

The last author kept all records and collected data, which was blinded from the first author, whose role was solely to perform lectures and exercises (Table 1). Data was collected at baseline (11/10/2023), at the end of the intervention period (15/11/2023), and three months post-intervention (14/02/2024).

**Table 1:** Breathing intervention program.

Week	Subject of lecture	Techniques	Home practice (20 minutes daily)
1	Measurements	Coherent breathing	Coherent breathing
	Short introduction		
2	Overview breath	Coherent breathing with focus on nose	Coherent breathing with focus on nose
	Breath and feelings	Breathhold technique	Alternate nasal breathing
	Change of habits	Nose opening smile	Nosebreathing when walking
	Nasal function:	Humming	Saline nose rinsing
	Why nasal breathing matters	Alternating nasal breathing	Two minutes breath awareness
	Anatomy and physiology	Two minutes breath awareness	Occational taping of mouth (day/night)
	Principles of nasal rehabilitation		
3	Muscular function:	Basic movements connected to breathing	Coherent breathing with focus on diafraghm
	Respiratory musculary system	Coherent breathing with focus on	and/ or stretching
	Functions of the diafraghm	respiratory musculature	As week 2
4	The autonomous nervous system (ANS):	Coherent breathing	Coherent breathing
	How the ANS works	Alternate nasal breathing	As week 2 plus sigh of relief
	How breath affects ANS	Sigh of relief	
	Respiratory Sinys Arythmia		
	Hearth Rate Variation		
5	Gas exchange and acid/base balance:	Coherent restrictive breathing	Coherent restrictive breathing
	The physiology of gas exchange,	Restrictive breathing (appr. 80%	Alternating nasal breathing
	hyperventilation and repiratory drive	of normal volume)	
6	Measurements		
	Feasibility assessment		

### Measures

The Nijmegen Questionnaire (NQ), previously used for hyperventilation syndrome and now validated for DB, was administered to quantify DB symptoms [41]. The NQ consists of 16 questions ranked on a five-point Likert scale where “never” counts as 0 and “very often” counts as 4, giving a total DB score between 0–64. Healthy, asymptomatic individuals generally have a DB score between 7–12. A score above 19 is suggestive of DB [41].

The Subjective Health Complaints Inventory (SHC) was used to measure the participants’ general health. This validated instrument measures the full range of subjective health problems [42]. The form lists 29 common somatic and psychological ailments, where the degree of complaints and duration must be stated for the last 30 days, graded on a four-point scale. The 29 individual health issues were grouped into five sub-issues [musculoskeletal pain (8 items), pseudoneurology (7 items), gastrointestinal disorders (7 items), allergic disorders (5 items), colds (2 items)], and a total score comprising all items. All scales were scored 0–100 (where 0 indicates no symptom pressure and 100 the highest possible symptom pressure).

The Measure Yourself Medical Outcome Profile (MYMOP) was initially published in 1996 [43] and revised to MYMOP-2 after a second validation [44]. The MYMOP questionnaire has been extensively evaluated and is a responsive and valid instrument [43–45]. Participants are asked to rate four items (symptom 1, symptom 2, well-being, and impact of symptoms on their daily activity status) on a scale of 0–6 where 0 is “As good as it could be” and 6 is “As bad as it could be”. Participants report one or two symptoms (physical or mental) which bother them the most, consider how bad each symptom was during the last week, and score them accordingly. They also report how much these symptoms affected a particular activity, their well-being during the last week, use of medication, and any possible adverse effects or worsening of symptoms. The symptom pressure and general well-being (scales 0–6) are presented.

End-tidal CO_2_ (EtCO_2_) (Microstream^TM^) is an objective measure of hyperventilation and is easily detected through a nasal cannula with a capnograph together with respiratory rate (RR). The normal values of EtCO_2_ are 4.7 kPa - 6 kPa [46]. Values below 4.6 are regarded as hypocapnia. The measurement was performed with the patient sitting relaxed while three measurements at 30-second intervals were conducted. Mean EtCO_2_ is presented. Respiration rates were registered simultaneously.

The registrations of HRV were performed using HeartMath with the participants in a sitting position, relaxed, and breathing regularly. HeartMath was chosen for HRV measurement as the instrument is cost-effective, and the ear-clip sensor is user-friendly for collecting HRV data. The system also performs well in recording and storing data. In the 1990s, HeartMath Institute researchers identified a physiological state called heart coherence [47]. Physiologically, the coherence state is marked by the development of a smooth, sine-wave-like pattern in the heart rate variability trace. This characteristic pattern, called heart rhythm coherence, is the primary indicator of the psychophysiological coherence state. The emWave Coherence score is a measure of the degree of coherence in the heart rhythm pattern. A coherent heart rhythm is a stable, regular, repeating rhythm resembling a sine wave at a single frequency between 0.04–0.24 Hz (3–15 cycles per minute). The scoring algorithm continuously monitors the most current 64 seconds of heart rhythm data and updates the score every 5 seconds. The more stable and regular the heart rhythm frequency, the higher the coherence score. Scores range from 0–16 [48].

Blood pressure was measured using the Beurer blood pressure monitor BM 28, with the participant sitting relaxed. Three measurements were performed on the left arm, and mean systolic and diastolic values were presented.

In addition to the aforementioned measures, participants were asked to fill in a diary with their daily exercises and their reactions to these.

### Sample size

The findings of this trial will be used to calculate the sample size for a potential future RCT. A study size of n = 15 participants was considered sufficient to generate adequate data [49].

### Statistical analyses

Data were analysed using paired samples t-tests and Wilcoxon paired tests. Mean and standard deviation (SD), standard error of the mean (SE), and 95% confidence interval of the mean (95% CI) are presented. The internal reliability of the different scales was assessed through the calculation of Cronbach’s alpha coefficients. To address dropout and non-responders, an intention-to-treat analysis was planned. Missing values at the end of the intervention (6 weeks) and three months post-intervention were imputed using multiple imputations. The multiple imputation was performed using a fully conditional specification model, applying linear regression as the prediction method for scale variables and two-way interactions for categorical variables. Five complete datasets with 10 iterations per dataset were generated. The combined estimates are presented as mean (SD) values. Due to the small sample size, bootstrapping with 1000 samples was performed to calculate 95% CI for the assumed population. All analyses were performed using SPSS v.29.0. All tests were two-sided. P values <0.05 were considered statistically significant.

## Results

A total of 18 individuals were referred to the trial by local GPs over a period of three weeks. Three individuals declined participation due to work or family commitments. Fifteen individuals provided informed consent to participate and were included for data registration. Three participants withdrew during the programme due to illness and attendance difficulties, resulting in an attrition rate of 20%. At three months post-intervention, 11 patients registered questionnaire data (attrition rate = 27%).

### Characteristics of the participants

The participants comprised 12 women [mean (SD) age 50.8 (9.4) years, range 39–74 years, with a mean (SD) NQ-score of 31.4 (5.2) points] and 3 men [mean (SD) age 46.5 (6.4) years, range 42–51 years, with a mean (SD) NQ-score of 30.0 (6.3) points].

Internal reliability tests showed acceptable Cronbach’s alpha values for the NQ-scale (0.727), SHC musculoskeletal pain (0.665), SHC pseudoneurology (0.624), SHC gastrointestinal disorders (0.724), SHC allergic disorders (0.577), SHC colds (0.665), and SHC total score (0.840).

### Dysfunctional breathing

The intervention appears to have produced beneficial results, both one week after the intervention and three months post-intervention. In particular, the NQ-scores (scale 0–64), indicating the level of disturbed breathing, show positive results both 6 weeks post-intervention (95% CI 3.2–9.9 points, p < 0.001) and three months post-intervention (95% CI 4.9–9.8 points, p < 0.001) compared to baseline. The imputed data set consisted of five imputed data sets with full data on all 15 included participants. Mean (SE) values from the pooled dataset are shown in Table 2.

**Table 2:** Results from a 5-week pilot breathing therapy program (baseline, end program, and three months post-program). Imputed data set (n = 15). Pooled mean values (standard error of the mean).

	Baseline		One week post-intervention		Three months post-intervention		p-value (baseline-one week post- intervention)	p-value (baseline-three months post- intervention)
NQ-score^1^	31.1	(1.4)	24.6	(2.0)	23.9	(1.5)	<0.001	<0.001
MYMOP symptom score^2^	4.2	(0.3)	2.7	(0.4)	3.4	(0.4)	<0.001	0.114
MYMOP wellbeing score^2^	4.1	(0.3)	2.7	(0.3)	2.7	(0.3)	<0.001	<0.001
SHC musculosceletal pain^3^	46.4	(4.6)	37.4	(4.2)	27.9	(3.7)	0.032	<0.001
SHC pseudoneurology^3^	43.2	(3.4)	37.0	(4.7)	35.1	(3.4)	0.130	0.028
SHC gastrointestinal problems^3^	27.0	(4.2)	30.7	(4.5)	20.1	(3.1)	0.188	0.006
SHC allergy^3^	19.1	(4.2)	15.5	(3.5)	13.3	(3.5)	0.161	0.038
SHC flu^3^	22.2	(5.6)	27.2	(7.3)	12.3	(4.7)	0.528	0.147
SHC total score^3^	35.8	(3.0)	32.5	(3.4)	24.4	(3.0)	0.073	<0.001
Systolic blood pressure (mm/Hg)	129	(4.3)	136	(4.3)	133	(3.4)	0.063	0.394
Diastolic blood pressure (mm/Hg)	85	(1.6)	87	(2.0)	82	(1.9)	0.145	0.142
EtCO_2_^4^	3.8	(0.2)	3.6	(0.3)	4.4	(0.2)	0.666	0.028
HRV^5^	2.6	(0.4)	2.5	(0.4)	2.3	(0.3)	0.840	0.587

1. Nijmegen questionnaire (scale 0–64. highest = worst); 2. Measure Yourself Medical Outcome Profile (scale 0–6. highest = worst); 3. Subjective Health Complaints (scale 0–100. highest = worst); 4. End Tidal CO_2_; 5. Heart Rate Variability – Coherent breathing

### General well-being

The MYMOP symptom pressure score (scale 0–6) was reported to be lower at the end of the intervention (95% CI 0.9–2.1 points, p < 0.001) but does not maintain statistical significance at three months (p = 0.114). The MYMOP well-being score (scale 0–6), considered a proxy for general quality of life, shows positive results both after six weeks (95% CI 0.7–2.2 points, p < 0.001) and three months (95% CI 0.7–2.1 points, p < 0.001) compared to baseline.

### General health

The SHC musculoskeletal pain score (scale 0–100) is also reported lower at six weeks (95% CI 0.8–17.2 points, p = 0.032) and is further decreased at three months (95% CI 11.1–25.3 points, p < 0.001). The SHC pseudoneurology complaints (scale 0–100) were not significantly altered at six weeks (p = 0.130) but improved at three months (95% CI 0.9–15.3 points, p = 0.028), as with the three-month scores of SHC gastrointestinal problems and SHC allergy scores (scales 0–100) (95% CIs 2.0–11.8 points, p = 0.006; 0.3–11.4 points, p < 0.038, respectively). The total symptom burden, as measured by the SHC total scores (scale 0–100) was not significantly altered at six weeks (p = 0.073) but improved at three months (95% CI 7.7–15.1 points, p < 0.001).

The EtPCO_2_ value was not significantly altered at six weeks (p = 0.666) but improved at three months (95% CI −1.1 - −0.1 points, p < 0.028).

These findings reflect a rather small sample (n = 15) and to simulate the results of the intervention in a larger sample or population we performed bootstrapping with 1000 randomly selected samples (Table 3). The starting point for the bootstrapping was the original data file and the bootstrapped data revealed, as expected, a more conservative picture. However, the NQ-score, the HSC well-being score, the SHC musculoskeletal pain score, and the SHC total symptom pressure score were improved after three months.

**Table 3:** Results from a 5-week pilot breathing therapy program (baseline, end program, and three months post-program). Original data set. Bootstrapped (1000 samples), mean (standard deviation) values, 95% confidence interval for the mean difference between baseline and three months post-intervention.

	Original data set						Bootstrap values				
	Baseline (n = 15)		One week post-intervention (n = 12)		Three months post-intervention (n = 11)		p-value (baseline-one week post- intervention)	p-value (baseline- three months post- intervention)	95% CI for mean difference. baseline to three months post- intervention		
NQ-score^1^	31.1	(5.2)	24.8	(8.4)	23.8	(6.3)	0.056	0.027	(2.4	–	8.7)
MYMOP symptom pressure score^2^	4.2	(1.3)	2.8	(1.4)	3.4	(1.6)	0.027	0.670	(−1.7	–	1.9)
MYMOP wellbeing score^2^	4.1	(1.1)	2.7	(1.2)	2.7	(1.0)	0.011	0.003	(<0.1	–	2.1)
SHC musculosceletal pain^3^	46.4	(17.7)	37.9	(18.1)	27.7	(1.0)	0.193	0.014	(10.1	–	25.6)
SHC pseudoneurology^3^	43.2	(13.0)	36.9	(20.3)	34.8	(16.2)	0.159	0.190	(−4.1	–	21.1)
SHC gastrointestinal problems^3^	27.0	(16.3)	30.6	(19.0)	19.9	(13.8)	0.783	0.050	(4.1	–	15.6)
SHC allergy^3^	19.1	(16.3)	15.6	(14.9)	12.7	(15.5)	0.079	0.680	(−3.8	–	4.8)
SHC flu^3^	22.2	(21.5)	27.8	(31.3)	12.1	(21.2)	0.380	0.050	(9.5	–	42.9)
SHC total score^3^	35.8	(11.6)	32.4	(14.6)	24.5	(13.5)	0.038	0.010	(7.5	–	15.8)
Systolic blood pressure (mm/Hg)^4^	129	(16.5)	135	(18.6)	132	(16.5)	0.549	0.736	(−12.3	–	9.0)
Diastolic blood pressure (mm/Hg)^4^	85	(6.1)	87	(8.4)	82	(9.0)	0.612	0.380	(−3.8	–	11.7)
EtCO_2_^4, 5^	3.8	(0.9)	3.6	(1.2)	4.4	(0.3)	0.948	0.180	(−1.3	–	−0.1)
HRV^4, 6^	2.6	(1.6)	2.4	(1.4)	2.3	(1.1)	0.183	0.168	(−0.1	–	3.0)

1. Nijmegen questionnaire (scale 0–64. highest = worst); 2. Measure Yourself Medical Outcome Profile (scale 0–6. highest = worst); 3. Subjective Health Complaints (scale 0–100. highest = worst); 4. n = 9; 5. End Tidal CO_2_; 6. Heart Rate Variability – Coherent breathing.

### Results feasibility

The application of the NQ as a tool for the identification of potential study participants was considered an easy and effective method by the involved GPs. The recruitment speed was three weeks and resulted in 18 potential participants from two GP practices, which was considered a feasible recruitment speed and practical to implement in a busy workday at a GP practice.

Three patients (17%) declined participation due to work and family commitments, resulting in 15 participants in the trial. Three of fifteen participants (20%) withdrew from the trial due to illness, migraine, and hospitalisation. Of the remaining twelve participants, three missed each one of the five clinical sessions due to a common cold, indicating a total program adherence of 95%. It should be noted that the intervention was performed during the winter season when, in addition to COVID-19, the common cold is a usual occurrence.

Most participants (n = 9) completed their diaries, but some (n = 4) found the rigidity of diary writing tiresome. Some participants felt that the breathing techniques were difficult to practise, as illustrated by the following citation from one participant’s diary:

*“Overall, I think I am getting the hang of the breathing exercises and that they help. But there are still times when I feel like I can’t fully ‘breathe out.’ Sometimes, I feel like I am breathing too fast for several days in a row, and when I do the breathing exercises then, I can’t find a flow or calmness and improvement, rather it feels more chaotic and like too much breathing.”*

When asked at one week post intervention, the participants expressed a desire for more written material illustrating the breathing exercises. At three months, they requested more active follow-up after the end of the intervention. According to data from the diaries, most of the participants (n = 5) had practised daily; however, verbally, all participants reported that they had continued their daily breathing exercises.

Participants’ oral reflections on the intervention after three months were solely positive, especially emphasising the importance of a group setting and getting to know the other participants.

The intervention programme was considered safe as no adverse effects were reported by the participants.

## Discussion

This pilot trial aimed to determine the short-term (6 weeks) and longer-term (three months) impact of a 5-week breathing therapy programme. After 6 weeks, we found improvements in dysfunctional breathing (DB), lower symptom scores, a higher degree of general well-being, and improved musculoskeletal pain. After three months, improvements were observed in DB, general well-being, and musculoskeletal pain. Additionally, lower degrees of pseudoneurological, gastrointestinal, and allergy complaints, as well as lower general symptom scores, were found. Moreover, after three months, we observed improved end-tidal CO_2_ values among participants. When data were bootstrapped to simulate a larger sample, positive results were found after three months regarding dysfunctional breathing, general well-being, musculoskeletal pain, and total symptom scores.

The trial also aimed to explore patient adherence and attrition rates. Three of the fifteen participants withdrew from the programme (refused to participate), giving an attrition rate of 20%, which is considered satisfactory.

The breathing therapy aimed to improve breathing efficiency and correct breathing parameters associated with dysfunctional breathing, such as hypocapnia, increased breathing rate, abnormal breathing patterns and mouth breathing, with the goal of improving musculoskeletal pain and other symptoms commonly associated with MUPS [14]. The improvements in symptoms observed in this trial align with earlier studies showing reduced symptoms and improved quality of life in MUPS patients after breathing and relaxation therapy [23]. The total symptom pressure, as measured by responses to 29 different health complaints and symptoms, showed a significant reduction both after the intervention and three months post-intervention.

Quality of life and/or well-being represent internal, subjective perceptions of aspects of general health such as vitality, pain, anxiety and depressive symptoms [50]. Measures of an individual’s general well-being are particularly useful in clinical research as they can detect the patient’s real-life experience as well as psychological disorders, including anxiety and depression [51]. Our results also showed significant improvement in general well-being both after one week and three months post-intervention. This is consistent with other studies on breath therapy [52,53].

The breathing programme in our trial emphasised the importance of establishing nasal breathing as a foundation for functional breathing. Nasal breathing enhances oxygenation, supports CO_2_ retention, and supports slower, deeper breathing patterns with a prolonged expiratory phase [54]. These effects have been associated with the physiological relaxation response [55–57]. As elaborated by Noble and Hochman (2019), recent studies in rodents and humans have directly linked slow nasal respiration to slow brain rhythms. In rodents, these are dissociable from other low-frequency rhythms and modulate local gamma activity in the medial prefrontal cortex, organising prefrontal network activity [58]. The positive results associated with nasal breathing may thus affect certain brain regions involved in emotion regulation, which may have treatment implications for stress management and anxiety [52,56,59,60].

In addition to nasal breathing, the programme emphasised resonance breathing with diaphragmatic (low) breathing. This technique has been shown to support autonomic balance via vagal stimulation [54–56],as measured through HRV [39]. The observed improvements in wellbeing and reduction in symptom burden may, in part, be attributed to this exercise [20,23,38,39]. Interviews conducted at week 18 and breathing diary notes indicated that most participants continued daily breathing exercises post intervention, coherent nasal breathing being the most frequently used technique.

The breathing exercises used in this trial aimed to normalise levels of EtPCO_2_. The breathing exercises also intended to normalise end-tidal CO_2_ (EtCO_2_) levels. Prior research has described an inverse relationship between nasal resistance and EtCO_2_ [54], and it is likely that reduced stress and improved autonomic balance, supported by both nasal and coherent breathing, contributed to less hyperventilation and reduced hypocapnia. An improvement in EtPCO_2_ was noted but not until the three months post intervention. The initial lack of significant improvements after five weeks of breathing training indicates that a prolonged training period may be needed to normalise biochemical measures of dysfunctional breathing. This is supported by other research showing that normalisation of CO_2_ is not achieved rapidly [61–62]. Moreover, it indicates that researchers should take this time frame into account when designing future studies.

### Feasibility considerations

We consider that the mode of recruitment was adequate, and that recruitment from GP practices and using the Nijmegen questionnaire (score ≥ 20) as inclusion criteria was promising. Attrition between baseline measurements and three months post intervention is to be expected. The somewhat high attrition rate (27%) found in this trial may be reduced in a later RCT through a participant-centric approach [63] through building incentives (e.g., one-to-one discussion of results and progress forward, travel reimbursements), reminders (email, phone), and more active follow-up (weekly digital educational videos).

Participants’ emphasised the importance of a group setting and the importance of getting to know the other participants. A group based approach supporting the development of a shared social identity among recipients [64] may be of importance in a later RCT but also when implementing breathing therapy in general practice. Qualitative data (participant feedback and follow-up support) should be measured and assessed for relevance in later iterations of the breathing programme.

Neither HRV nor BP revealed significant improvements after three months. The HRV and BP were measured in a clinical setting, which may have affected the results, e.g., giving a ‘white coat effect’ [65]. The use of portable HRV monitors, wearable actigraphs, and 24-hour blood pressure readings may identify improvements in these measures obliterated by the “white coat effect.

The subjective measurements, e.g., the questionnaires, showed significant improvements, indicating that these measures may be of use in a later RCT. However, the use of subjective measurements may induce bias, e.g., reference bias, recall bias and social desirability bias, and a later trial should thus combine such subjective measurements with objective measurements such as portable HRV monitors, wearable actigraphs, and 24-hour blood pressure readings.

We would also note that a three months follow up is a rather short period of time, and we suggest that a later trial should seek to prolong the follow up period to assess sustainability of the the results.

### Strengths and limitations

The results from this pilot and feasibility trial are not representative as it consists of a very low number of participants; as such, the results and conclusions cannot be generalised to any other setting. In addition, the lack of a RCT-design limits the generalizability of the results, and also causal attribution (e.g., placebo effects, natural symptom fluctuations). We also acknowledge the general limitations of HRV as a measurement tool, as it is influenced by several confounding variables such as sleep, caffeine intake, food consumption, cognitive activity, posture, and susceptibility to movement artifacts [66]. Moreover, this study did not implement standardization protocols to control for these confounding factors. We also acknowledge the limitations of using the emWave Coherence Score, as it may not be directly comparable to HRV indices used in other studies employing standardized time-domain or frequency-domain analyses. However, the purpose was to form the basis for a larger RCT and evaluate the feasibility of the trial parameters, such as trial procedures, the validity of tools, the estimation of the recruitment rate, and the estimation of parameters and calculating sample size for a larger RCT designed to assess the effect of a breathing programme for MUPS [67].

## Conclusion

A five-week programme of weekly breathing exercises and education showed beneficial results on several patient-reported outcomes such as dysfunctional breathing, general well-being, and musculoskeletal pain. Additionally, lower degrees of pseudoneurological, gastrointestinal, and allergy complaints, as well as a lower general symptom burden, were found. Moreover, after three months, we found improved end-tidal CO_2_ values among participants. The feasibility of the programme, regarding recruitment, inclusion, attrition and participant engagement was satisfactory. We thus find that the programme is promising and that a randomised controlled trial is needed to calculate the effect of this intervention.
